# Disruption to Transformation: Aging in the New Normal: A Chat With NIA Senior Leadership

**DOI:** 10.1093/geroni/igab046.1043

**Published:** 2021-12-17

**Authors:** Melinda Kelley, Melinda Kelley

## Abstract

The National Institute on Aging (NIA) at the National Institutes of Health, Department of Health and Human Services, is the federally designated lead agency on aging research and supports significant research on aging as a lifelong process. In the last six years, NIA has experienced a tripling of its budget. Although much of this funding is targeted to Alzheimer’s disease (AD) and AD-related dementias research, there has been an increase in funds allocated to non-AD research in keeping with the overall growth of NIH. This symposium will provide a forum for exploration of the implications of the budget increases for the general research community. NIA’s senior staff will discuss research priorities and programs supported by the Institute. A question-and-answer session will follow these remarks on current funding and future priorities and research directions of NIA.

